# Prospective study of daily low-dose nedaplatin and continuous 5-fluorouracil infusion combined with radiation for the treatment of esophageal squamous cell carcinoma

**DOI:** 10.1186/1471-2407-9-408

**Published:** 2009-11-22

**Authors:** Satoshi Osawa, Takahisa Furuta, Ken Sugimoto, Takashi Kosugi, Tomohiro Terai, Mihoko Yamade, Yasuhiro Takayanagi, Masafumi Nishino, Yasushi Hamaya, Chise Kodaira, Takanori Yamada, Moriya Iwaizumi, Kosuke Takagaki, Ken-ichi Yoshida, Shigeru Kanaoka, Mutsuhiro Ikuma

**Affiliations:** 1 First Department of Medicine, Hamamatsu University School of Medicine, Hamamatsu, Japan; 2 Center for Clinical Research, Hamamatsu University School of Medicine, Hamamatsu, Japan; 3 Department of Radiology, Hamamatsu University School of Medicine, Hamamatsu, Japan; 4 Department of Molecular Diagnosis, Hamamatsu University School of Medicine, Hamamatsu, Japan

## Abstract

**Background:**

Protracted low-dose concurrent chemotherapy combined with radiation has been proposed for enhanced treatment results for esophageal cancer. We evaluated the efficacy and the toxicity of a novel regimen of daily low-dose nedaplatin (cis-diammine-glycolatoplatinum) and continuous infusion of 5-fluorouracil (5-FU) with radiation in patients with esophageal squamous cell carcinoma.

**Methods:**

Between January 2003 and June 2008, 33 patients with clinical stage I to IVB esophageal squamous cell carcinoma were enrolled. Nedaplatin (10 mg/body/day) was administered daily and 5-FU (500 mg/body/day) was administered continuously for 20 days. Fractionated radiotherapy for a total dose of 50.4-66 Gy was administered together with chemotherapy. Additional chemotherapy with nedaplatin and 5-FU was optionally performed for a maximum of 5 courses after chemoradiotherapy. The primary end-point of this study was to evaluate the tumor response, and the secondary end-points were to evaluate the toxicity and the overall survival.

**Results:**

Twenty-two patients (72.7%) completed the regimen of chemoradiotherapy. Twenty patients (60.6%) achieved a complete response, 10 patients (30.3%) a partial response. One patient (3.0%) had a stable disease, and 2 (6.1%) a progressive disease. The overall response rate was 90.9% (95% confidence interval: 75.7%-98.1%). For grade 3-4 toxicity, leukopenia was observed in 75.8% of the cases, thrombocytopenia in 24.2%, anemia in 9.1%, and esophagitis in 36.4%, while late grade 3-4 cardiac toxicity occurred in 6.1%. Additional chemotherapy was performed for 26 patients (78.8%) and the median number of courses was 3 (range, 1-5). The 1-, 2- and 3-year survival rates were 83.9%, 76.0% and 58.8%, respectively. The 1- and 2-year survival rates were 94.7% and 88.4% in patients with T1-3 M0 disease, and 66.2% and 55.2% in patients with T4/M1 disease.

**Conclusion:**

The treatment used in our study may yield a high complete response rate and better survival for each stage of esophageal squamous cell carcinoma.

**Trial registration:**

ClinicalTrials.gov Identifier: NCT00197444

## Background

Results of a series of clinical trials indicate that definitive chemoradiotherapy (CRT) for esophageal cancer produces more promising results than radiation therapy alone, and is considered to be the standard treatment for patients with medically inoperable or surgically unresectable esophageal cancer [1-3]. Recently reported results obtained with this treatment indicate it can provide survival benefits comparable to those in the Western series of surgery alone, and is one of the standard treatments, even for resectable-stage disease [4]. In Japan, where squamous cell carcinoma is dominant in esophageal cancer, the use of CRT rather than surgery is spreading, thus making it desirable to establish a more effective CRT protocol to achieve a complete response rate and improved survival. It is also necessary to investigate which anticancer drugs are more efficacious and how to best use these drugs in combination with radiation for better complete response rates and survival [5].

Nedaplatin (cis-diammine-glycolatoplatinum: CDGP) is a second-generation platinum complex that was developed to reduce nephrotoxicity and maintain the effectiveness of cisplatin [6-8]. Numerous single agents have been tested for the treatment of esophageal cancer and the overall response rate has typically ranged from 15%-30% [9,10], whereas the response rate for nedaplatin as a single agent was 51.7% with little toxicity in a phase II study [11]. Recently, phase I and II studies of chemoradiotherapy using intermittent standard-dose nedaplatin with 5-fluorouracil (FU) for esophageal squamous cell carcinoma were performed and demonstrated the safety and efficacy of this treatment [12-14].

Protracted low-dose concurrent chemotherapy combined with radiation has been proposed for more satisfactory local control rates without severe systemic toxic effects [15,16]. Platinum is not only a cytotoxic agent, but also a chemical modulator and radiosensitizer that enhances the chemotherapeutic effects of 5-FU on tumor cells [17-19]. Therefore, daily low-dose nedaplatin and 5-FU combined with radiation may be a more effective regimen than the previously reported intermittent standard-dose regimen.

In this study, we prospectively evaluated the efficacy and the toxicity of a regimen of daily low-dose nedaplatin and continuous infusion of 5-FU combined with radiation in patients with esophageal squamous cell carcinoma at an institution in Japan.

## Methods

### Patients and pre-treatment evaluation

Between January 2003 and June 2008, 33 patients with histologically proven squamous cell carcinoma of the esophagus were enrolled and treated in accordance with our protocol. Eligibility criteria were: Eastern Cooperative Oncology Group performance status 0-2; age <85 years; white blood cells >3 × 10^3^/μl; platelets >1 × 10^5^/μl; serum total bilirubin <2.0 mg/dl; serum transaminase <3 times the upper normal limit; serum creatinine <1.5 mg/dl; creatinine clearance ≥ 60 ml/min; no serious cardiac disease; no prior chemotherapy or radiotherapy and receipt of informed consent.

The tumor stages were classified according to the TNM classification (sixth edition) of the International Union against Cancer (UICC). Tumor stages were conventionally determined by means of computed tomography (CT) or magnetic resonance imaging (MRI) of the neck, chest and abdomen, endoscopy and esophagography. Endoscopic ultrasonography was performed to determine the tumor invasion within the esophageal wall for the patients with suspected Stage I disease. As a rule, patients with disease limited to the mucosal layer and those with metastasis to distant organs were excluded from this study, but patients who had distant lymph node metastasis that could be encompassed in a single radiation field were included [M1 lymph node metastasis (M1 lym)].

### Treatment

Low-dose nedaplatin (10 mg/body/day) was administered daily for 20 days on days 1-5, 8-13, 15-19 and 22-26, and 5-FU (500 mg/body/day) was continuously administered for 20 days on days 1-5, 8-13, 15-19 and 22-26. A serotonin receptor antagonist was preventively given as an antinauseant just before the administration of nedaplatin.

A linear accelerator (6 MV or 10 MV) was used as the X-ray source for the external radiotherapy. Positioning of the fields and dosimetry were studied using a CT scan and 3D treatment planning. The treatment fields encompassed the tumor bed with 3-5 cm proximal and distal margins and 1 cm lateral margins. Supraclavicular nodes were included in the treatment portals for the upper and the middle thoracic tumors; celiac nodes were included for the lower and the middle thoracic tumors. Fractionated external radiotherapy was performed from the first day of chemotherapy, administrated 5 days a week, and a total dose of 50.4-66 Gy was delivered at a rate of 1.8-2.0 Gy per fraction to all but two patients. After a dose of 40 Gy, the field was changed for all but two patients to avoid the spinal cord irradiation, and only macroscopic lesions were irradiated with a margin of at least 1 cm. Two patients with stage I disease and diagnosed before March 2005 were given 12 Gy/3 fractions of high-dose-rate intraluminal brachytherapy (HDRIBT) after 40 Gy of external irradiation. HDRIBT was performed at a level 5 mm below the surface of the mucosa with a margin of at least 2 cm by remote after loading system every 3-4 days; the total planned dose of external and intraluminal irradiation was 52 Gy. Dose variation of radiation therapy among the enrolled patients was presented in Table 1.

**Table 1:** Variations of initial irradiation dose

Radiation therapy	Dose variation	Patients
**External and intraluminal **	40 Gy + 12 Gy	2*
**External**	50.4 Gy	2
	60 Gy	19
	66 Gy	8

*, who were diagnosed as clinical Stage I (T1N0 M0)

Chemotherapy interruption criteria were: white blood cell count <2 × 10^3^/mm^3^; platelet count <5 × 10^4^/mm^3^; body temperature of 38°C or more and any other life-threatening toxicities. Irradiation interruption criteria were a white blood cell count of <1.5 × 10^3^/mm^3 ^and any other life-threatening toxicities. The study protocol was approved in advance by the Human Institutional Review Boards of Hamamatsu University School of Medicine and was registered as ClinicalTrials.gov Identifier: NCT00197444. Written informed consent was obtained from all patients before starting treatment. This study was conducted to conform to the principles of the Declaration of Helsinki.

For patients who showed an objective response to the treatment, additional chemotherapy was optionally performed using 14 mg/m^2^/day nedaplatin and continuous infusion of 500 mg/m^2^/day 5-FU on days 1-5. This chemotherapy was repeated every 4 weeks for a maximum of 5 courses, after which no further treatment was performed if a complete response was obtained.

### Evaluations of response and toxicity

The primary end-point of this study was to evaluate the tumor response, the response rate (RR) and the complete response (CR) rate. The secondary end-points were to evaluate the toxicity and the overall survival. Follow-up evaluations were performed by endoscopy with biopsy, and CT and/or MRI of the neck, chest and abdomen, every 3 months for the first year and every 6 months thereafter. All the follow-up data were updated at the end of December 2008. Tumor response was assessed according to the Response Evaluation Criteria in Solid Tumors (RECIST) (UICC, 2002) and initially evaluated within 1 month after chemoradiotherapy. RECIST does not refer to CR criteria for primary lesions by endoscopy in detail, and endoscopic methods of evaluation have not yet been fully validated. In this study, CR for the primary tumor was defined by endoscopy when all visible tumors, including ulceration, disappeared with negative biopsy and lasted for ≥ 4 weeks according to the previous studies [20,21]. Confirmation of CR and PR were usually evaluated 3 months after initial evaluation. Toxicities were graded according to the Common Terminology Criteria for Adverse Events v3.0. All of the early hematologic toxicities were defined to be related with chemoradiotherapy and observed before additional chemotherapy.

### Statistical analysis

Statistical differences in tumor response rates between the 2 groups were determined by Fisher's exact test. The Kaplan-Meier method was used for survival calculations based on the first day of chemoradiotherapy, and the log-rank test for comparisons between groups. All *p*-values were two-sided, and a level of *p *< 0.05 was considered to be statistically significant. All statistical analyses were performed using the StatView software for Windows, Version 5.0 (SAS Institute, Cary, NC, USA).

## Results

### Patient characteristics

The demographic clinical characteristics of the 33 patients are presented in Table 2. The median age was 66.0 years (range, 55-82 years). Most of the patients had tolerable performance status for the treatment; the performance status was 0, 1 and 2 in 6, 21 and 6 patients, respectively. According to TNM classification, there were 7, 3, 12 and 11 patients with T1, T2, T3 and T4 disease, respectively. Lymph node metastasis was observed in 23 patients. Among these patients, there were 6 patients with M1 lym. Nineteen patients had T1-3 M0 diseases that were in resectable stages, whereas 14 patients had T4 and/or M1 lym disease. The median follow-up period for all patients was 19 months (range, 6-66 months). No patient was lost to follow-up.

**Table 2:** Patient characteristics

No. of patients	33
**Male/Female**	25/8

**Age, years: median (range)**	66 (55--82)

**Performance status (ECOG)**	
0/1/2	6/21/6

**Stage (UICC 1997)**	
I*	6
IIA/B	3/3
III	15
IVA/B	3/3

**TNM clinical classification (UICC 1997)**	
T1/T2/T3/T4	7/3/12/11
N0/N1	10/23
M0/M1 lym	27/6

**Pathology**	
Well/Mod/Poor	4/25/4

**Location**	
Upper/Middle/Lower	3/19/11

ECOG, Eastern Cooperative Oncology Group; UICC, International Union Against Cancer; M1 lym, M1 lymph node metastasis; Well, well-differentiated squamous carcinoma; Mod, moderately-differentiated squamous carcinoma; Poor, poorly-differentiated squamous carcinoma

*: disease with submucosal invasion

### Response to therapy

For the initial response, 20 of the 33 patients achieved a complete response (CR), 10 had a partial response (PR), 1 had stable disease (SD) and 2 had progressive disease (PD). The overall response rate (CR + PR) was 90.9% (95% confidence interval [CI] = 75.7%-98.1%). The CR rate for T1-3 M0 disease was significantly higher than for T4/M1 disease (84.2% versus 28.6%; *p *= 0.0031), while no statistical significance was observed for the response rate (CR + PR). The CR rate was significantly higher in the absence than in the presence of lymph node metastasis (90.0% versus 47.8%, *p *= 0.0495), while again no statistical significance was observed in the response rate (Table 3).

**Table 3:** Response results

Category	**Total No**.	CR	PR	SD	PD	RR (%)
**Overall**	33	20	10	1	2	90.9
						
T1-3 M0	19	16	3	0	0	100
T4/M1	14	4	7	1	2	78.6
						
N0	10	9	1	0	0	100
N1	23	11	9	1	2	87.0

CR, complete response; PR, partial response; SD, stable disease; PD, progressive disease; RR, response rate

### Survival rate

**Figure 1:**
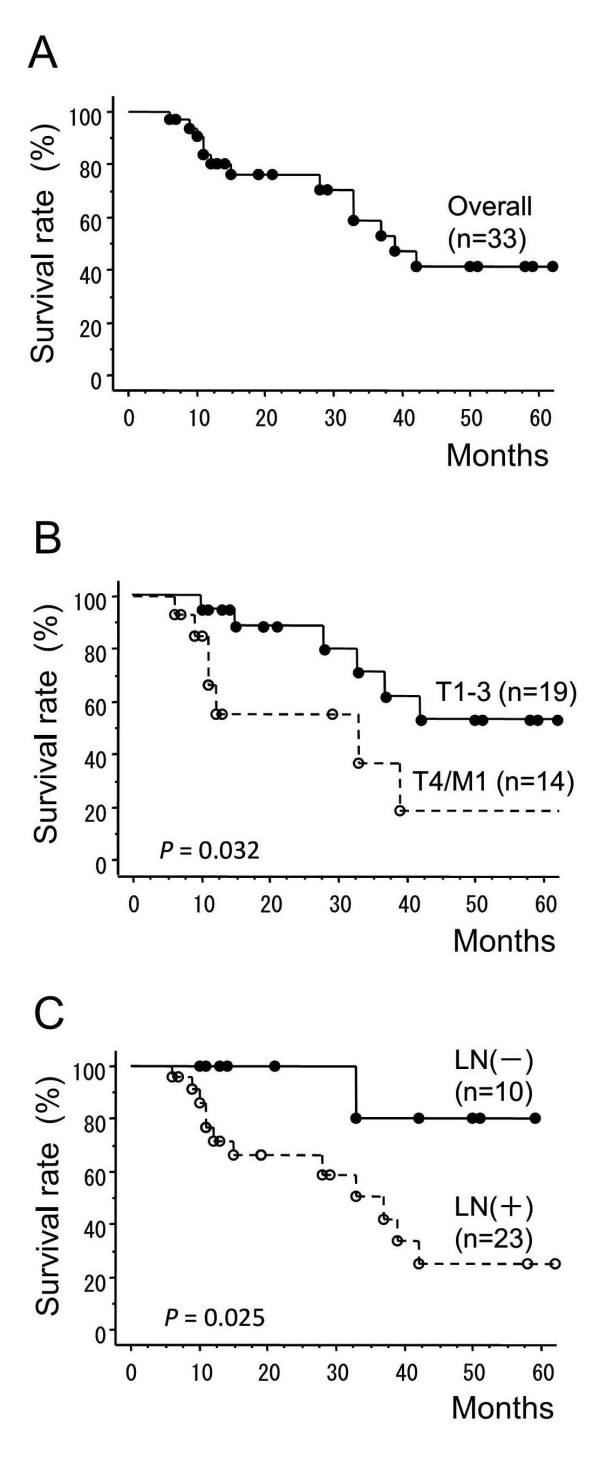
**Survival rate calculated by the Kaplan-Meier method**. (A) Overall survival among all patients. (B) Comparison between T1-3 M0 and T4/M1 groups. (C) Comparison between groups of patients with and without lymph node metastasis.

Thirteen patients had died by the end of follow-up. Salvage surgery after recurrence was not performed in any of the patients in this series. The overall survival rates at 1, 2 and 3 years were 83.9% (95% CI = 71.0%-96.8%), 76.0% (95% CI = 60.3%-91.7%) and 58.8% (95% CI = 37.8%-79.8%). respectively. Median survival time (MST) was 39.0 months (95% CI = 29.5%-48.5%) (Figure 1A). We compared the survival rates of the 19 patients with T1-3 M0 disease with those of the 14 patients with T4/M1 disease. The 1- and 2-year survival rates were 94.7% (95% CI = 84.7%-100.0%) and 88.4% (95% CI = 73.2%-100.0%) respectively, in patients with T1-3 M0 disease and were 66.2% (95% CI = 38.8%-93.6%) and 55.2% (95% CI = 25.0%-85.4%) respectively, in patients with T4/M1 disease. The survival rates between T1-3 M0 and T4/M1 patients were significantly different (*p *= 0.032) (Figure 1B). A comparison of the survival rates of 10 patients without lymph node metastasis with those of 23 patients with lymph node metastasis also showed a significant difference (*p *= 0.025) (Figure 1C). The 1- and 2-year survival rates for patients without lymph node metastasis were both 100%, and for those with lymph node metastasis they were 76.7% (95% CI = 58.9%-94.5%) and 66.1% (95% CI = 45.5%-86.7%), respectively.

### Toxicity

The toxicity grades detected in this study are shown in Table 4. Grade 3-4 leukopenia was observed in 25 patients (75.8%) and grade 3-4 thrombocytopenia in 8 patients (24.2%). As for non-hematological toxicity, grade 3-4 esophagitis was found in 12 patients (36.4%). As for late toxicity, grade 3-4 pericarditis was detected in 2 patients and grade 3 pleuritis in 1 patient. No treatment-related deaths occurred in this study.

**Table 4:** Toxicities

	Grade					
		
Toxicity	0	1	2	3	4	Grade ≥3 (%)
*~Early toxicity~*						
Leukopenia	0	1	7	21	4	75.8
Thrombocytopenia	1	13	11	8	0	24.2
Anemia	3	15	12	2	1	9.1
Renal dysfunction	32	1	0	0	0	0
Transaminase	33	0	0	0	0	0
Esophagitis	1	11	9	12	0	36.4
Nausea/Vomiting	17	11	5	0	0	0
Mucositis	23	5	3	2	0	6.1
*~Late Toxicity~*						
Pericarditis	24	6	1	1	1	6.1
Pneumonitis	11	21	0	0	0	0
Pleuritis	24	8	0	1	0	3.0
Esophageal stricture	27	0	1	5	0	15.2
Bone fracture	32	0	1	0	0	0

### Effect of protocol completion or additional chemotherapy on survival rate

**Figure 2:**
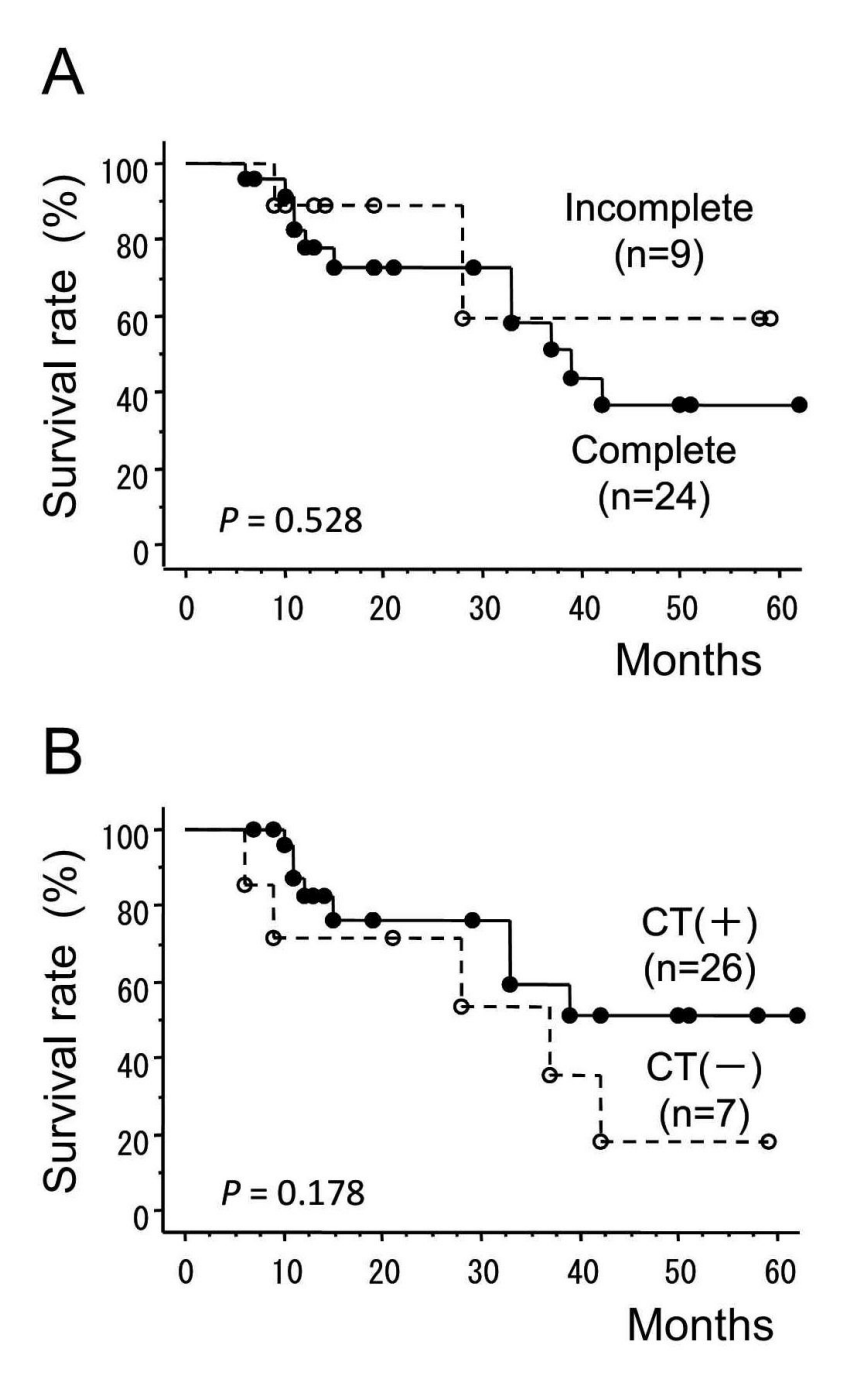
**The effects of treatment completion or additional adjuvant chemotherapy on survival rate**. (A) Comparison between groups that did and groups that did not complete the treatment. (B) Comparison between groups with or without additional chemotherapy after the initial chemoradiotherapy. CT, chemotherapy.

Nine patients (27.3%) did not complete the regimen of chemoradiotherapy because of adverse events in the acute phase. Among these, one patient did not complete both of the radiation therapy (at the dose of 36 Gy) and chemotherapy due to severe bone marrow suppression and febrile condition. Another patient did not complete the radiation therapy at the dose of 46 Gy due to the Grade 3 leukopenia and patient's refusal of the continuation of therapy due to depressive mental condition. The other 7 patients could not continue the chemotherapy suffered from hematological toxicities. We examined whether the protocol completion affected the survival rates. There was no statistically significant difference in survival rates between those who completed chemoradiotherapy and those who did not (Figure 2A).

In the present study, additional optional chemotherapy with nedaplatin and 5-FU, repeated every 4 weeks for a maximum of 5 courses, was performed for patients showing an objective response to the treatment. Additional chemotherapy was performed for 26 patients (78.8%), and the median number of courses was 3 (range, 1-5). Although the number of patients was small and the follow-up period was relatively short in this series, there was no statistically significant difference in survival rate between the patients who received additional adjuvant chemotherapy and those who did not (Figure 2B).

## Discussion

Protracted low-dose concurrent chemotherapy combined with radiation is one of the methods which have been proposed for improve tumor response and survival of esophageal cancer patients [5]. In this study, we prospectively evaluated the efficacy and toxicity of a regimen of daily low-dose nedaplatin, a second-generation platinum, and continuous infusion of 5-FU combined with radiation in patients with esophageal squamous cell carcinoma at an institution in Japan. The overall response rate (90.9%) and MST (39.0 months) for this treatment were relatively high compared with other chemoradiotherapy regimens for esophageal cancer. The 1- and 2-year survival rates were 94.7% and 88.4%, respectively, in patients with T1-3 M0 disease and 66.2% and 55.2%, respectively, in patients with T4/M1 disease. The treatment used in our study can thus be expected to yield superior survival rates for every stage of esophageal squamous cell carcinoma.

Since the Radiation Therapy Oncology Group (RTOG) phase III trial (RTOG 85-01) and the inter-group phase III trial, concurrent chemoradiotherapy using 5-FU and cisplatin has been considered the standard treatment for locally advanced esophageal cancer [1,3,22]. For this therapy, the chemotherapy agents are administered intermittently. According to previous clinical trials of this type of chemoradiotherapy for esophageal squamous cell carcinoma, T1N0 M0 patients showed 3- and 5-year overall survival rates of 80% and 75.5%-77%, respectively, while the corresponding rates in T2-3/M0 patients were 49% and 46%, with a MST of 34 months [23]. In T4/M1 patients, 3- and 5-year overall survival rates 22%-23% and 13%-17%, with a MST of 9-10 months [21,24]. This type of chemotherapy regimen is characterized by a significant incidence of high-grade acute gastrointestinal and renal toxicity.

Low-dose protracted infusion chemotherapy combined with radiotherapy has been used for several cancers in the hope of attaining a longer radiosensitizing effect without severe toxicity, and positive results have been reported [25-27]. However, only a few studies have been conducted of low-dose protracted infusion chemotherapy combined with radiotherapy for oesophageal cancer [28-30]. A recent randomized phase II study of esophageal cancer by the Japanese Radiation Oncology Study Group, which used protracted low-dose cisplatin and continuous 5-FU infusion, did not indicate any superiority of survival over standard intermittent chemotherapy combined with radiation [30]. In the study presented here, we used nedaplatin, which is reportedly a more effective anticancer drug for oesophageal cancer when used alone, for reducing gastrointestinal and renal toxicity. Details of previous clinical studies of chemoradiotherapy using nedaplatin and 5-FU for esophageal cancer are shown in Table 5. For definitive chemoradiotherapy using intermittent standard-dose nedaplatin and 5-FU regimens, the CR rate was reported to be 9%-73% with 1- and 2-year survival rates of 30.7%-65.1% and 10.2%-45.9%, respectively. Compared to the intermittent standard-dose nedaplatin and 5-FU regimens, the daily low-dose nedaplatin and 5-FU regimen seems to yield relatively high CR and survival rates. However, leukopenia was also frequently observed in patients receiving the daily low-dose regimen, even though the total drug doses were almost the same. Esophageal cancer surgery is still the standard treatment for resectable stages. Recent studies have shown that neoajuvant chemotherapy or chemoradiotherapy with surgery can provide survival advantages for locally advanced esophageal cancer [31,32]. However, recovery of health-related quality of life with surgery is reportedly significantly reduced compared to that with definitive chemoradiotherapy [33,34], since the latter has the absolute advantage of conserving the esophagus and stomach. A certain number of patients are therefore likely to choose this treatment until a more effective methodology is developed.

**Table 5:** Comparison among chemoradiotherapy studies, using nedaplatin/5-FU for esophageal cancer

A. Daily low-dose nedaplatin and 5-FU regimens								
**Author**	**No**.	**Radiation** **(Gy)**	**Total dose (mg)** **Nedaplatin** **5-FU**	**Stage**	**RR** **(%)**	**CR** **(%)**	**Survival rate (%)**	**Leucopenia (%)**

Inaba H [37]	10	60	200/body 10,000/body	I--IVB	80	50	80 (1 y)	90

Osawa S *	33	50.4--66	200/body 10,000/body	I--IVB	90.9	60.6	83 (1 y) 77 (2 y)	75.8

**B. Intermittent standard-dose nedaplatin and 5-FU regimens**								

**Author**	**No**.	**Radiation** **(Gy)**	**Total dose (mg)** **Nedaplatin** **5FU**	**Stage**	**RR** **(%)**	**CR** **(%)**	**Survival rate (%)**	**Leucopenia (%)**

Kato H [38]	22	60--66	160/m^2^ 5,000/m^2^	I--IV	77	9	30.7 (1 y) 10.2 (2 y)	15.4
		
	17	40	80/m^2^ 25,000/m^2^	pre-S II--IV	70.6	28.6	48.2 (1 y) 12.1 (2 y)	

Yamanaka H [39]	17	40	200/body 14,000/body	I--IVB	76.5	11.8	52.9 (1 y)	17.6

Nemoto K [40]	17	60--70	200/body 15,000/body	I--IVA	94.1	41.2	59 (1 y) 39 (2 y)	25
		
	7	60--70	200/body 15,000/body	post-S recur	100	0	69 (1 y) 69 (2 y)	

Ishikura S [14]	26	60	180/m^2^ 8,000/m^2^	III--IVB	---	12	50 (1 y) 31 (2 y)	35

Sato Y [12]	26	60	100/m^2^ 4,000/m^2^	I--IVA	88.5	42.3	65.1 (1 y) 37.2 (3 y)	40

Kodaira T [13]	40	60	360/m^2^ 10,500/m^2^	III--IV	76	48	58.9 (1 y) 45.9 (2 y)	80

Yamashita H [41]	12	50.4	160/m^2^ 6,400/m^2^	II--IVB	82	73	40 (1 y) 13 (2 y)	50

Jingu K [42]	30	60	140/m^2^ 5,000/m^2^	post-S recur	73.3	13.3	60.6 (1 y) 56.3 (3 y)	30

*, present study; RR, response rate; CR, complete response; pre-S, pre-surgical; post-S recur, post-surgical recurrence; 1 y, 1-year; 2 y, 2-year

Although previous studies of intermittent standard-dose chemoradiotherapy using cisplatin and 5-FU reported 2-10% treatment related death [3,22], no treatment-related death occurred in our study in spite of frequent early hematological toxicity. One of the advantages of protracted low-dose concurrent chemotherapy combined with radiation is that we could discontinue the chemotherapy when severe adverse effects such as bone marrow suppression were observed during the treatment, and we could also restrict the total doses of anticancer drugs to minimize these adverse effects. Our results show that failure to complete the protocol was not associated with survival, but may have contributed to the absence of treatment-related death. Long-term cardiac toxicity and pleural effusion after CRT for esophageal cancer are significant problems for quality of life and future survival. During our treatment, the frequency of pericarditis and/or pleuritis seemed to be similar to that in previously reported studies using intermittent standard-dose cisplatin and 5-FU [35,36].

Although the protocol of this study had not any restriction of the salvage surgery for locoregional recurrence and we were always worth considering the salvage surgery after recurrence, there was no patient in this series who was able to tolerate to and agree to the salvage surgery due to circumstances. Therefore, all of the patients who had locoregional recurrence were treated with chemotherapy in this series. If the salvage surgery would be more safe treatment option for the high risk patients and its indication would be expanded, further improvement of survival shall be expected.

There are several limitations to this study. We allowed the dose of radiation to vary from 50.4-66 Gy and also administered intraluminal brachytherapy after 40 Gy of external irradiation in 2 patients. These variations may have affected the response or survival rates, although the RTOG 94-05 trial made it clear that higher radiation doses (64.8 Gy) could not improve locoregional control compared to that obtained with 50.4 Gy [22]. We also permitted optional additional chemotherapy that could be repeated for a maximum of 5 courses after chemoradiotherapy. We performed a subgroup analysis for patients treated with adjuvant chemotherapy, which confirmed there was no statistically significant advantage in the survival curve. However, we may not conclude that our results completely deny the value of adjuvant chemotherapy, since the number of patients was small and follow-up period may not be sufficient.

Because of the relatively small number of patients in our study, further clinical studies are required with a larger number of patients and in a multicenter setting to confirm whether daily low-dose nedaplatin and continuous infusion of 5-FU combined with radiation is an effective alternative to the conventional or modified RTOG 85-01 regimen using intermittent standard doses of cisplatin and 5-FU. Nevertheless, our results indicate that the former may provide a higher CR rate and survival for patients with esophageal squamous cell carcinoma. We believe this regimen is a promising candidate meriting a phase III trial to determine whether it can become the standard regimen for esophageal squamous cell carcinoma.

## Conclusion

Daily low-dose nedaplatin and continuous 5-FU infusion combined with radiation may yield a higher CR rate and better survival for patients with esophageal squamous cell carcinoma.

## Abbreviations

CDGP: cis-diammine-glycolatoplatinum (nedaplatin); 5-FU: 5-fluorouracil; CRT: chemoradiotherapy; UICC: International Union against Cancer; CT: computed tomography; MRI: magnetic resonance imaging; M1 lym: M1 lymph node metastasis; HDRIBT: high-dose-rate intraluminal brachytherapy; CI: confidence interval; MST: Median survival time; RTOG: Radiation Therapy Oncology Group.

## Competing interests

The authors declare that they have no competing interests.

## Authors' contributions

SO conducted this study and wrote the manuscript. TF, KS and MI contributed to the study design and coordination. TK contributed to the planning of irradiation. TT, MY, YT, MN, YH, CK, TY, MI, KT, KY, and SK performed the chemoradiotherapy and follow-up. All the authors have read and approved the final manuscript.

## Pre-publication history

The pre-publication history for this paper can be accessed here:

http://www.biomedcentral.com/1471-2407/9/408/prepub
